# A DyP-Type Peroxidase of *Pleurotus sapidus* with Alkene Cleaving Activity

**DOI:** 10.3390/molecules25071536

**Published:** 2020-03-27

**Authors:** Nina-Katharina Krahe, Ralf G. Berger, Franziska Ersoy

**Affiliations:** Institut für Lebensmittelchemie, Gottfried Wilhelm Leibniz Universität Hannover, Callinstraße 5, 30167 Hannover, Germany; rg.berger@lci.uni-hannover.de (R.G.B.); franziska.ersoy@lci.uni-hannover.de (F.E.)

**Keywords:** alkene cleavage, aryl alkenes, basidiomycota, biocatalysis, carotene degradation, dye-decolorizing peroxidase (DyP), manganese, *Komagataella pfaffii*, *Pleurotus sapidus*

## Abstract

Alkene cleavage is a possibility to generate aldehydes with olfactory properties for the fragrance and flavor industry. A dye-decolorizing peroxidase (DyP) of the basidiomycete *Pleurotus sapidus* (PsaPOX) cleaved the aryl alkene *trans*-anethole. The PsaPOX was semi-purified from the mycelium via FPLC, and the corresponding gene was identified. The amino acid sequence as well as the predicted tertiary structure showed typical characteristics of DyPs as well as a non-canonical Mn^2+^-oxidation site on its surface. The gene was expressed in *Komagataella pfaffii* GS115 yielding activities up to 142 U/L using 2,2′-azino-bis(3-ethylbenzthiazoline-6-sulphonic acid) as substrate. PsaPOX exhibited optima at pH 3.5 and 40 °C and showed highest peroxidase activity in the presence of 100 µM H_2_O_2_ and 25 mM Mn^2+^. PsaPOX lacked the typical activity of DyPs towards anthraquinone dyes, but oxidized Mn^2+^ to Mn^3+^. In addition, bleaching of *β*-carotene and annatto was observed. Biotransformation experiments verified the alkene cleavage activity towards the aryl alkenes (*E*)-methyl isoeugenol, *α*-methylstyrene, and *trans*-anethole, which was increased almost twofold in the presence of Mn^2+^. The resultant aldehydes are olfactants used in the fragrance and flavor industry. PsaPOX is the first described DyP with alkene cleavage activity towards aryl alkenes and showed potential as biocatalyst for flavor production.

## 1. Introduction

Many small aromatic aldehydes and ketones are volatiles with olfactory properties and therefore of high interest to the fragrance and flavor industry [1]. One method to generate aldehydes and ketones is the oxidative cleavage of alkenes. Chemical options are ozonolysis, dihydroxylation followed by oxidative glycol cleavage, or metal-based methods [2,3,4]. However, all of these methods have disadvantages, such as the generation of explosive intermediates, the use of environmentally unfriendly and/or toxic oxidants and metal catalysts, or low yield and low chemoselectivity [2]. An alternative is the application of enzymes due to their high chemo-, regio-, and stereospecificity as well as the possibility to use mild reaction conditions [3]. Another advantage is the generation of “natural” flavors according to effective legislation in Europe and the US. This becomes more and more important considering the rising popularity of natural products [5]. Different proteins of different enzyme classes, which are heme, non-heme iron, or non-iron metal dependent and have different protein structures as well as different reaction mechanism are known to catalyze alkene cleavage reactions [3]. Specifically, an isoeugnol and *trans*-anethole oxygenase from *Pseudomonas putida* and two manganese dependent enzymes from *Thermotoga maritima* (manganese-dependent Cupin TM1459) and *Trametes hirsuta* (Mn^3+^-dependent proteinase A homologue) oxidatively cleaved the benzylic double bond of different aryl alkenes, such as isoeugenol and *trans*-anethole to form the respective aldehydes [6,7,8,9]. In addition, alkene cleavage activity towards aryl alkenes was also detected for several peroxidases. Cleavage of different styrene derivatives was described for *Coprinus cinereus* peroxidase and a human myeloperoxidase as minor side reaction [10], while horseradish peroxidase (HRP) showed a chemoselectivity of 92% for the conversion of *trans*-anethole (90%) to *p*-anisaldehyde [11]. Furthermore, transformations of *o*-ethylisoeugenol and *trans*-anethole to the corresponding benzaldehyde derivatives by lignin peroxidases were described [3,11]. However, to our best knowledge no alkene cleavage activity of a dye-decolorizing peroxidase (DyP) is known.

DyP-type peroxidases (EC: 1.11.1.19) are a new superfamily of heme peroxidases that oxidize various dyes, in particular xenobiotic anthraquinone dyes, which are hardly oxidized by other peroxidases [12]. Furthermore, typical peroxidase substrates, such as ABTS (2,2′-azino-bis(3-ethylbenzthiazoline-6-sulphonic acid) and phenolic compounds are also substrates for DyPs [13,14]. However, amino acid sequences, protein structures, and catalytic residues differ highly between DyPs and other classes of heme peroxidases [15]. Typical structural characteristics of DyPs are the ferredoxin-like fold, which is formed by two domains containing α-helices and four-stranded antiparallel *β*-sheets, and a GXXGD motif [14,15]. The active site (heme pocket) including a catalytic aspartic acid and arginine over the heme plane (distal) and proximal histidine is structurally similar to other heme peroxidases, even though other peroxidases contain histidine instead of aspartic acid [16]. The proximal histidine in the heme pocket functions as the fifth ligand of the heme iron, while the distal aspartic acid and arginine are involved in the activation of the enzyme [14,15]. The deprotonated aspartic acid (or asparagine) mediates the rearrangement of a proton from hydrogen peroxide after it enters the heme pocket in the resting state. This results in the heterolytic cleavage of hydrogen peroxide to water and oxidation of the heme to the radicalic-cationic oxoferryl species Compound I by two-fold single electron transfer [16]. Even though the distal arginine is not directly involved in the rearrangement of hydrogen peroxide, it is essential for the coordination of hydrogen peroxide to the heme iron and the stabilization of Compound I [16]. During the following reaction cycle, Compound I is reduced by oxidation of two substrate molecules to the state during enzyme resting state in two sequential steps with Compound II as intermediate. However, the existence of Compound II has not been confirmed universally for all DyP-type peroxidases [17]. In the presence of excessive hydrogen peroxide suicide inhibition was observed for different DyPs [18,19]. This is also well known for classical peroxidases as a result of an inactive oxidative state (Compound III) and results from reaction of hydrogen peroxide with Compound II [20,21].

The objective of the present study was to identify new enzymes of basidiomycetes with alkene cleavage activity towards aryl alkenes. A screening was performed using *trans*-anethole as model alkene. A new DyP-type peroxidase from *P. sapidus* was semi-purified and the coding gene was identified. Heterologous expression resulted in the production of soluble protein and allowed the biochemical characterization of the DyP. The enzyme was able to oxidize Mn^2+^, but did not catalyze the degradation of anthraquinone dyes, which is typical for other DyPs. Biotransformation experiments verified the cleavage activity towards different alkenes. This is the first study describing a DyP with alkene cleavage activity towards aryl alkenes.

## 2. Results and Discussion

### 2.1. Purification and Identification of the Alkene Cleavage Activity

**Figure 1:**
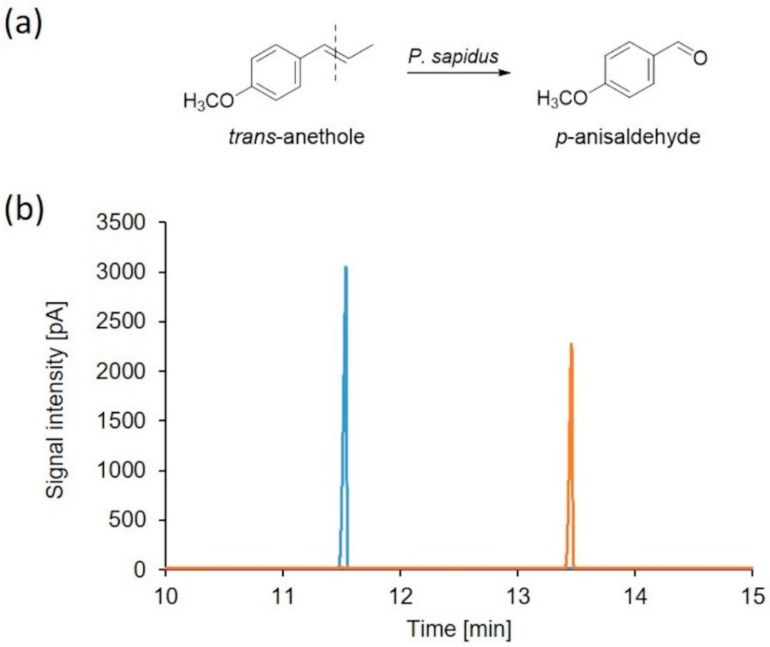
Bioconversion of *trans*-anethole by *P. sapidus*. (**a**) Alkene cleavage of *trans*-anethole resulted in the formation of *p*-anisaldehyde. (**b**) GC-FID chromatogram of an *n*-hexane extract of the conversion of the blank sample (blue) and after incubation with lyophilized mycelium of *P. sapidus* (orange). Retention times: *trans*-anethole (11.53 min) and *p*-anisaldehyde (13.45 min).

Within a screening of 17 basidiomycetes for alkene cleavage activity using the substrate *trans*-anethole (Table S1) *P. sapidus* turned out to be a promising candidate for the production of the desired activity. The lyophilized mycelium as well as the culture supernatant was examined for the ability to cleave *trans*-anethole after submerged cultivation. The culture supernatant showed no activity, whereas the incubation in the presence of the mycelium resulted in formation of 5.36 mM *p*-anisaldehyde (molar yield of 79.03%; Figure 1).

For the identification of the enzyme catalyzing the *trans*-anethole cleavage, it was semi-purified from the rehydrated mycelium by hydrophobic interaction and anion exchange chromatography (IEX). During the purification, a high activity loss occurred, which resulted in low product concentrations after conversion (Table S2). This was most likely a result of protein loss, enzyme degradation or denaturation as described for other enzymes [22,23,24]. Another possibility is the loss of cofactors or –substrates, such as metal ions or peroxides during the purification steps [6]. Addition of Mn^2+^ led to a 15-fold increase in *p*-anisaldehyde concentration, which was further increased by addition of hydrogen peroxide, indicating a cosubstrate dependency (Table S2). Chemical conversion by Mn^2+^ alone was excluded, while product formation was observed with hydrogen peroxide (18 µM), but with a yield around 30-fold lower than the one for the enzymatic reaction (Table S2). Thus, the improved bioconversion in the presence of Mn^2+^ and hydrogen peroxide was verified to be the result of an increased enzyme activity.

**Figure 2:**
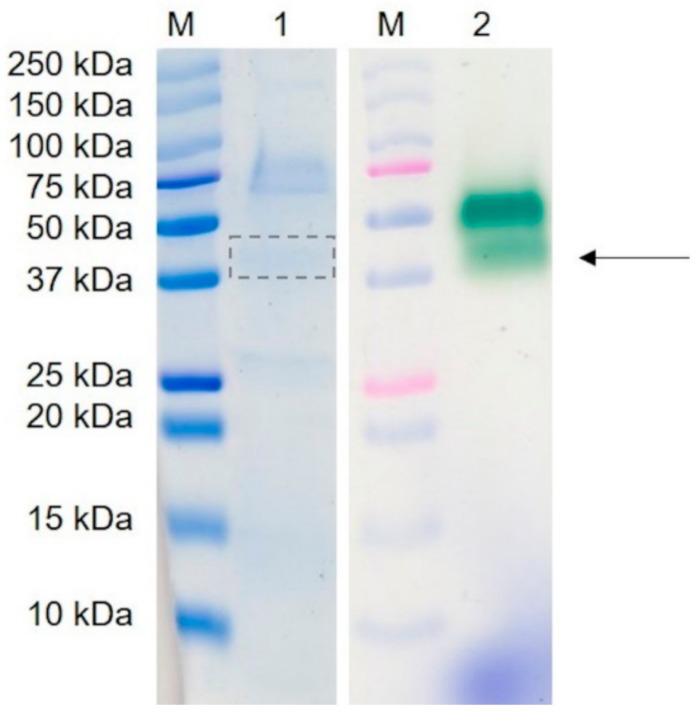
Semi-native PAGE of the active fraction after purification of the alkene cleavage enzyme from *P. sapidus* by IEX. 1: gel stained with Coomassie Brilliant Blue; 2: gel stained with ABTS in the presence of hydrogen peroxide, M: pre-stained molecular mass marker. An arrow and a box marks the protein that was successfully identified by sequencing.

A class of fungal enzymes that requires hydrogen peroxide and some of which need Mn^2+^ for catalysis are peroxidases [12,25,26]. The anion exchange fractions, which showed alkene cleavage activity, also exhibited peroxidase activity, thus verifying the presence of a peroxidase. For visualization of the activity, a semi-native PAGE was performed and stained with ABTS in the presence of hydrogen peroxide (Figure 2). Two peroxidases running at 45 and 52 kDa were detected. The respective protein bands stained with Coomassie Brilliant Blue were excised for electrospray ionization tandem mass spectrometry. Due to the low protein concentration of the 52 kDa band, no meaningful peptides were found (Figure 2, lane 1). This paper presents the data obtained for the protein running at 45 kDa (Figure 2, arrow).

Three tryptic peptides (EGSELLGAR, DGSFLTFR, and SGAPIEITPLKDDPK) were identified by ESI-MS/MS. Homology searches against the public database NCBI using the mascot search engine (Matrix Science, London, UK) identified a DyP-type peroxidase of *P. ostreatus* PC15 (*Pleos*-DyP4 [27]; GenBank accession no. KDQ22873.1), a close relative of *P. sapidus*, as the best hit.

### 2.2. Amplification and Expression of PsaPOX

**Figure 3:**
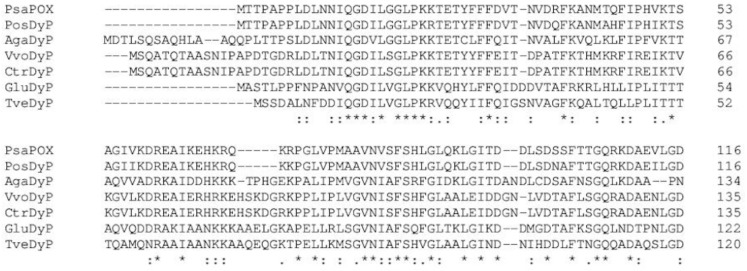

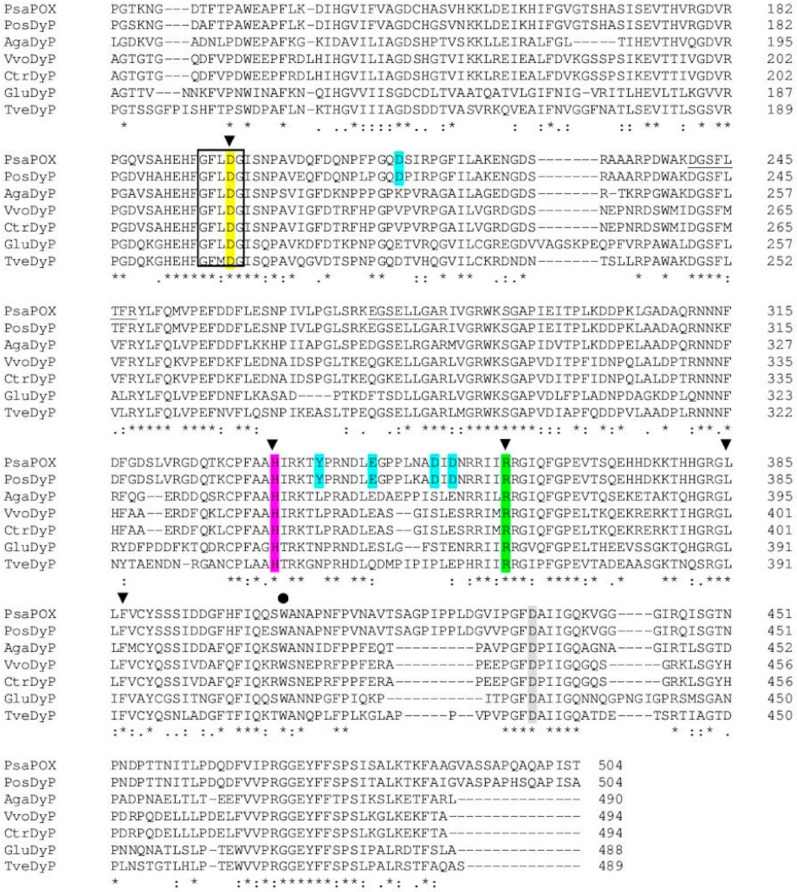
Alignment of alkene cleaving peroxidase from *P. sapidus* (PsaPOX) with the *Pleos*-DyP4 of *P. ostreatus* (PosDyP; KDQ22873.1) and other characterized DyPs. AgaDyP: *Armillaria gallica* (PBK80505.1), VvoDyP: *Volvariella volvacea* (AKU04643.1), CtrDyP: *Coriolopsis trogii* (AUW34346.1), GluDyP: *Ganoderma lucidum* (ADN05763.1), and TveDyP: *Trametes versicolor* (XP_008039377.1). Inverted triangles show amino acids important for heme binding (histidine (magenta) functions as ligand for heme and the four other amino acid residues form a hydrogen peroxide binding pocket). Aspartic acid, which forms a hydrogen bond with histidine to stabilize compound I (oxidized heme after transfer of two electrons to H_2_O_2_) is shown in grey. The black box indicates the GXXDG motif containing the catalytic aspartic acid residue (yellow), which cleaves H_2_O_2_ heterolytically with the help of the neighboring arginine (green) to form compound I, and the circle presents an exposed tryptophan potentially involved in an LRET (long range electron transfer). Important amino acids for Mn^2+^-oxidation are highlighted in cyan; asterisks indicate conserved residues, colons equivalent residues and dots partial residue conservation. Peptides identified by protein sequencing are underlined. Alignment was performed with Clustal Omega (European Bioinformatics Institute, Hinxton, UK).

Specific primers successfully amplified the 1512 bp coding region of the gene from *P. sapidus*. The translated amino acid sequence of 504 aa contained the peptide fragments that were obtained by ESI-MS/MS and showed highest identity (94%) to the sequence of *Pleos*-DyP4 (Figure 3), which has not been investigated for its alkene cleavage activity against *trans*-anethole or other substrates before [27,28]. In our hands, *P. ostreatus* showed a weaker *trans*-anethole cleavage activity, too (Table S1). Identity of the *P. sapidus* peroxidase (PsaPOX) to other DyPs and proteins was lower than 60%. A sequence alignment with other DyPs (Figure 3) confirmed that PsaPOX exhibited the typical GXXGD motif and all important residues known for the catalytic activity of DyPs, such as the proximal histidine (His-334) (fifth ligand of heme iron) and distal Asp-196 and Arg-360 involved in the activation of the enzyme (formation of compound I) by H_2_O_2_ cleavage [14,15]. Furthermore, Trp-405 was identified as a homolog to the surface exposed Trp-377 of AauDyP of *Auricularia auricula-judae*, which serves as an oxidation site for bulky substrates such as Reactive blue 19 (RBB19) using a long-range electron transfer [29]. Comparison of the PsaPOX and *Pleos*-DyP4 sequence indicated that PsaPOX exhibited a non-canonical Mn^2+^-oxidation site on its surface (Asp-215, Glu-345, Asp-352 and Asp-354; Trp-339 participates in the electron transport from the oxidation site to the heme) like *Pleos*-DyP4 [28] and can oxidize Mn^2+^ to Mn^3+^, which is known for a few fungal DyPs only [24,27,30,31].

A structural homology model of PsaPOX (Figure S1), which was generated using the X-ray crystal structure of *Pleos*-DyP4 (PDB-ID 6fsk) on the SWISS-MODEL server, possessed typical characteristics of the DyP-type peroxidase family (N- and C-terminal ferredoxin-like domain, each formed by four-stranded antiparallel *β*-sheets and several *α*-helices) [15] and supported the classification of PsaPOX as DyP. Furthermore, the analysis of the amino acid sequence using PeroxiBase [32] related the *P. sapidus* peroxidase (PsaPOX) to the class “DyP-type peroxidase D”. As known from literature, DyPs differ significantly in amino acid sequence, tertiary structure, and catalytic residues from other representatives of the heme peroxidases, such as HRP, human myeloperoxidase, lignin peroxidase, or *Coprinus cinereus* peroxidase [12,15,33], all of which are able to cleave *trans*-anethole or structurally related alkenes [10,11]. So far no DyP is known to catalyze the mentioned reaction.

### 2.3. Production and Purification of the Recombinant PsaPOX

The *PsaPOX* gene was amplified and cloned into the *K. pfaffii* expression vector pPIC9. The initial expression of the gene yielded average peroxidase activities of 65 U/L after 72 h of cultivation. The best performing colonies produced activities up to 142 U/L, indicating a multiple insertion of the expression construct [34]. Similar results were obtained for the heterologous production of a DyP from *Funalia trogii* in *K. pfaffii* previously [24]. Further experiments were performed using the clone with the highest peroxidase activity for maximum protein production.

**Figure 4:**
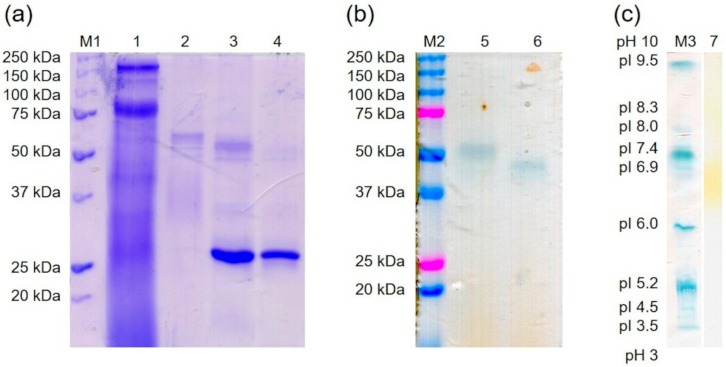
Purification of the recombinant PsaPOX by Ni-IMAC. (**a**) SDS-PAGE stained with Coomassie Brilliant Blue. 1: flow through, 2: elution fraction, 3: elution fraction incubated with EndoH, 4: EndoH, M1: molecular mass marker. (**b**) Semi-native PAGE stained with ABTS in the presence of hydrogen peroxide. 5: elution fraction, 6: elution fraction incubated with EndoH, M2: pre-stained molecular mass marker. (**c**) Isoelectric focusing gel. 7: elution fraction stained with phenylendiamine in the presence of urea peroxide. M3: standard protein marker for isoelectric focusing stained with Coomassie Brilliant Blue.

The recombinant peroxidase was purified by Ni-NTA affinity. Using SDS-PAGE, a molecular mass of around 61 kDa was determined (Figure 4a), which is slightly higher than the calculated molecular mass of 54.9 kDa (ExPASy). In addition, the native recombinant enzyme was detected at 52 kDa after semi-native PAGE, while the native wild-type enzyme showed a band at 45 kDa (Figure 2 and Figure 4b). Deglycosylation by endoglycosidase H (EndoH) showed that the higher molecular mass was attributed to post-translational modifications by *K. pfaffii*, as has been described for other proteins [35,36]. The wild-type peroxidase, on the contrary, was not glycosylated (see Figure 2 and Figure 4b). That is uncommon for DyPs, which usually exhibit a carbohydrate content of 9 to 30% [12]. The molecular mass of the monomeric PsaPOX was similar to other DyP-type peroxidases [12].

Analysis of the purified recombinant peroxidase by isoelectric focusing indicated an isoelectric point around pH 6.7 (Figure 4c), which differs slightly from the calculated value of 6.28 (ExPASy), but was similar to the isoelectric point of another DyP-type peroxidase from *P. sapidus* [19]. Most other proteins belonging to the DyP-type peroxidase family showed lower values (pI 3.5-4.3, [12]).

### 2.4. Biochemical Characterization of PsaPOX

**Figure 5:**
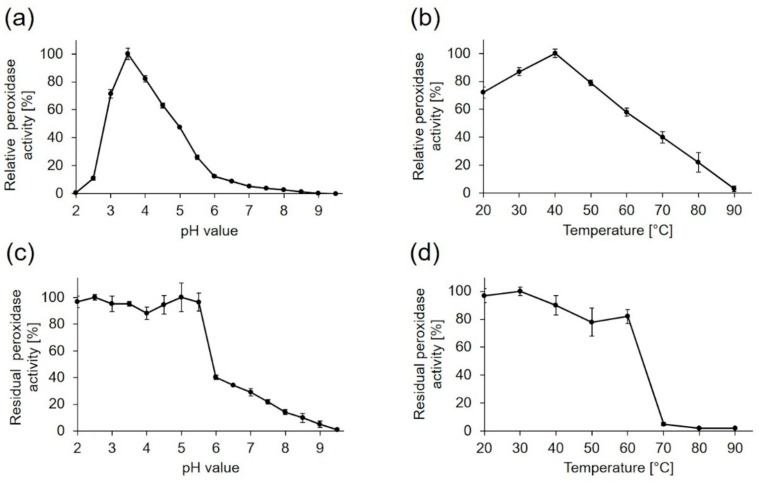
Influence of pH and temperature on activity and stability of PsaPOX. The pH optimum (**a**) was determined to be 3.5 and the temperature optimum (**b**) 40 °C. Relative peroxidase activity [%] was defined as the percentage of activity detected with respect to the highest activity in each experiment. pH stability (**c**) was determined after incubation of PsaPOX in Britton Robinson buffer ranging from pH 2.0 to 9.5 for 1 h at RT and temperature stability (**d**) after incubation at 20 to 90 °C and pH 3.5 for 1 h. Residual activities were determined at pH 3.5 and 40 °C. Values are the average of triplicate experiments with standard deviations shown as error bars.

The influence of pH and temperature on PsaPOX activity and stability was determined using ABTS in the presence of hydrogen peroxide as substrate (Figure 5). The enzyme showed a pH optimum of 3.5 while more than 50% of activity was conserved between pH 3 and 5 (Figure 5a). At lower or higher pH values of ≤ 25% of activity remained, most likely due to conformational changes of the enzyme. The results were consistent with the findings for other fungal DyPs, which had pH optima in the range between pH 2 and 5 [13,19,27]. PsaPOX showed the highest pH stability with a residual peroxidase activity of ≥90% between pH 2.0 and 5.5 after 1 h of incubation (Figure 5c). At pH values higher than six, near the isoelectric point, the stability decreased drastically, probably due to a reduced solubility and changes of the protein structure, which may have resulted in protein aggregation.

Peroxidase activity of PsaPOX increased with rising temperature, reaching its maximum at 40 °C (Figure 5b), which was similar to the optimum (30–40 °C) of a recombinant DyP from *P. ostreatus* [13], but higher than the optimum (RT) of another DyP-type peroxidase of *P. sapidus* produced heterologously in *Escherichia coli* [37]. With further temperature increase, the peroxidase activity of PsaPOX decreased continuously. The temperature stability of PsaPOX was determined after an incubation for 1 h at different temperatures (Figure 5d). The enzyme was relatively stable at temperatures from 20 to 60 °C with a residual activity ≥80%. At higher temperatures, a high loss of activity was observed due to protein denaturation, resulting in residual activities <5%. The temperature stability of PsaPOX was higher than the stabilities of DyPs from *Bjerkandara adusta* and *Auricularia auricular-judae*, which were produced heterologously in *E. coli* (residual activity ≥80% and <5% after 1 h at 20–50 °C and 60 °C, respectively [13]), and of a DyP from *P. sapidus* produced in *Trichoderma reesei* (residual activity ≥80% and <65% after 5 min at 15-45 °C and 50 °C, respectively [19]).

**Figure 6:**
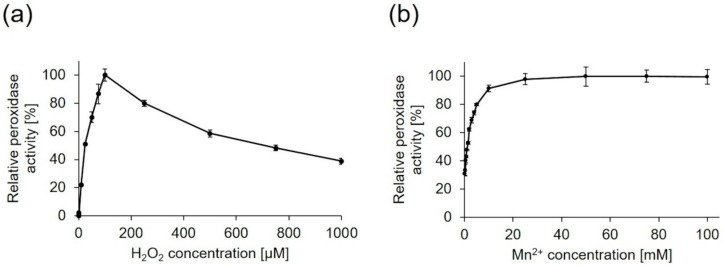
Effect of hydrogen peroxide (**a**) and Mn^2+^ concentration (**b**) on the activity of PsaPOX. Relative peroxidase activity [%] was defined as the percentage of activity detected with respect to the highest activity obtained in each experiment. Values are the average of triplicate experiments with standard deviations shown as error bars.

As mentioned above, the addition of hydrogen peroxide as well as Mn^2+^ led to an increase of the product concentration for the biotransformation of *trans*-anethole using the lyophilized mycelium of *P. sapidus* containing the wild-type PsaPOX (Table S2). For this reason, the hydrogen peroxide and Mn^2+^ dependencies were examined for the recombinant enzyme using ABTS as substrate at optimal pH and temperature (Figure 6). As expected, no peroxidase activity was detectable without hydrogen peroxide. The activity rose with increasing peroxide concentration and reached its optimum in the presence of 100 µM H_2_O_2_ (Figure 6a). An increase of the hydrogen peroxide concentration led to a continuous activity decrease. Suicide inhibition in the presence of excess hydrogen peroxide is well known for classical peroxidases as a result of the formation of an inactive oxidative state (Compound III) by reaction of H_2_O_2_ and Compound II [20,21], even if the existence of Compound II has not been confirmed for DyP-type peroxidases universally [17]. However, inhibition of other DyPs in the presence of higher hydrogen peroxide concentrations has been reported [18,19].

Investigation of the Mn^2+^ dependency (Figure 6b) showed that PsaPOX activity rose with increasing Mn^2+^ concentration, but was not completely dependent on the addition of Mn^2+^. 30% of peroxidase activity were detected without addition of Mn^2+^. PsaPOX reached the maximal activity in the presence of 25 mM Mn^2+^. Evaluation of Mn^3+^ formation by Mn^2+^ oxidation revealed a manganese peroxidase activity of 0.4 U compared to 1 U of peroxidase activity using ABTS as substrate. This result fits the prediction of a Mn^2+^ oxidation site. Only a few fungal DyPs are known to catalyze the oxidation of Mn^2+^ [24,27,30,31]. Calculation of kinetic constants (Table 1) showed that the catalytic efficiency of PsaPOX towards Mn^2+^ was similar to the one of *Pleos*-DyP1 from *P. ostreatus* and Ftr-DyP from *Funalia trogii* [24,27]. However, the catalytic efficiency of *Pleos*-DyP4 was higher [27].

Kinetic parameters were also calculated for the oxidation of ABTS at optimal conditions (Table 1). The affinity of PsaPOX to ABTS (37 µM) was similar to a DyP from *Irpex lacteus* (28 µM), but higher in comparison to the FtrDyP from *F. trogii* (182 µM) and the *Pleos*-DyP2 from *P. ostreatus* (787 µM) [18,24,27]. In contrast, the catalytic efficiency of PsaPOX (184 s^−1^ mM^−1^) was lower than the efficiency of the DyP from *Irpex lacteus* (8000 s^−1^ mM^−1^) and *Pleos*-DyP4 (352 s^−1^ mM^−1^), but higher than the efficiency of the FtrDyP (54 s^−1^ mM^−1^).

It is known that DyP-type peroxidases typically oxidize anthraquinones and other dyes. Exemplary, decolorization of Reactive blue 19 (anthraquinone dye) and Reactive black 5 (recalcitrant azo dye) by recombinant PsaPOX (1 U/L) was tested. Unexpectedly, PsaPOX showed activity for neither of the substrates (Table 1), although the protein sequence and tertiary structure as well as the presence of typical catalytic residues and the GXXDG motif identified the enzyme as a DyP-type peroxidase. However, *Pleos*-DyP1 from *P. ostreatus* and *Tv*DyP1 from *Trametes versicolor* also did not oxidize the high redox-potential Reactive black 5, although they degraded Reactive blue 19 [27,30]. A missing activity against Reactive blue 19 or another anthraquinone has not been described for a fungal or class D type DyP before, but one bacterial DyP of *Pseudomonas fluorescens* (DyP2B, DyP typ class B) is known not to oxidize the anthraquinone dye Reactive blue 4 [38].

### 2.5. Alkene Cleavage Activity of PsaPOX

To prove the ability of PsaPOX to convert *trans*-anethole to *p*-anisaldehyde, biotransformation experiments were performed at optimal conditions using 1 U/mL peroxidase activity. Substrate cleavage was detected in the presence of hydrogen peroxide (Table S3), whereas no activity was observed in its absence as expected from the peroxidase activity measurement with ABTS (Figure 6a). Due to the fact that the semi-purified wild-type DyP showed an alkene cleavage activity without addition of hydrogen peroxide (see Table S2) a low amount of H_2_O_2_ must have been present in the analyzed IEX fraction. This was verified by incubation of the fraction with *o*-dianisidine and HRP in the presence of *trans*-anethole. Oxidation of *o*-dianisidine by HRP, which requires H_2_O_2_ as cosubstrate, and formation of a red-brown reaction product occurred (Figure S2). Further, analysis of the IEF fraction regarding a hydrogen peroxide producing enzyme revealed a hypothetical protein from *P. ostreatus* (KDQ29984.1). It belongs to the glucose-methanol-choline (GMC) oxidoreductase family as evident from the best hit for the protein band at 75 kDa (Figure 2, lane 1) according to protein sequencing (identified tryptic peptides: AADLIK, AIAVEFVR, ELGGVVDTELR, AQYDAWAELNR, VADASIIPIPVSAHTSSTVYMIGER, DLASGDPHGVGVSPESIDVTNYTR, VLGGSTTINAMLFPR, EVVVSAGTIGTPK) and homology search against the public database NCBI (Figure S3). The protein contained seven of the eight tryptic peptides identified for the 75 kDa band. The last one was found with an amino acid exchange (Arg instead of Lys), which is most likely a result of the different fungal strains the proteins originate from. The protein from *P. ostreatus* showed >92% identity to another hypothetical GMC oxidoreductase from *P. ostreatus* and ≥55% identity to a glucose oxidase from *Moniliophthora roreri* and other fungal alcohol oxidases (Figure S3), which belong to the GMC oxidoreductase family and are known for the production of hydrogen peroxide during substrate oxidation. Thus, the oxidase (75 kDa band) most likely produced the detected hydrogen peroxide, which was subsequently used as cosubstrate by the wild-type PsaPOX. Due to the fact that the formation of *p*-anisaldehyde by the oxidase under production of H_2_O_2_ seemed highly unlikely and as no further oxidation products of *trans*-anethole were detected, *trans*-anethole was excluded as substrate. Instead, the buffer component Bis-Tris, which contains several alcohol groups, or carbohydrate functionalities of other proteins in the IEX fraction were assumed to be used as substrate by the oxidase.

As described for the wild-type DyP the *p*-anisaldehyde concentration increased for the biotransformation with the recombinant enzyme in the presence of 25 mM Mn^2+^ (Table S3). However, product formation in general was low. The residual peroxidase activity was determined during the biotransformation of *trans*-anethole (Figure S4). After 16 h, 62% of the activity remained, thus inactivation of the enzyme was not responsible for the relatively low product yields.

**Figure 7:**
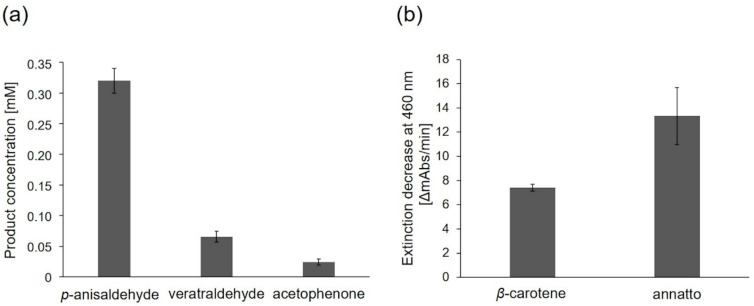
Alkene cleavage activity of PsaPOX on different substrates in the presence of 100 µM H_2_O_2_ and 25 mM MnSO_4_ at pH 3.5. (**a**) Product concentration after conversion of *trans*-anethole (6.7 mM) to *p*-anisaldehyde, (*E*)-methyl isoeugenol (6.7 mM) to veratraldehyde, and *α*-methylstyrene (6.7 mM) to acetophenone by PsaPOX (1 U/mL) at RT. The presented product concentrations are the differences between the values determined for the reaction with the active and heat inactivated enzyme (blank) (the original values are shown in Table S4). (**b**) Decolorization of 7% (*v/v*) *β*-carotene and 7% (*v/v*) annatto by PsaPOX (1 U/L) at 40 °C. Cleavage of carotenoids was shown as extinction decrease per min. Values are the average of triplicate experiments with standard deviations shown as error bars.

PsaPOX (1 U/mL) was further examined for alkene cleavage activity regarding other substrates in the presence of hydrogen peroxide and Mn^2+^. The aryl alkenes (*E*)-methyl isoeugenol as well as *α*-methylstyrene, which are derivatives of *trans*-anethole, were converted to the expected products (veratraldehyde and acetophenone), while piperine was not cleaved (Figure 7a). However, the resulting product concentration was fivefold lower for the biotransformation of (*E*)-methyl isoeugenol and more than tenfold lower for the conversion of *α*-methylstyrene than for *trans*-anethole. Different substrate specificities were also observed for the alkene cleavage by other peroxidases, such as HRP, *Coprinus cinereus* peroxidase, and a human myeloperoxidase [10,11], but a conversion of aryl alkenes using a DyP-type peroxidase has not been described before.

In addition to the described substrates, PsaPOX (1 U/L) also showed an alkene cleavage activity towards the natural dyes *β*-carotene and annatto (mixture of the xanthophylls bixin and norbixin), which was detected by substrate bleaching (Figure 7b). The activity for annatto was higher than for *β*-carotene. Cleavage of *β*-carotene and annatto is also known for other fungal DyPs [19,39,40,41]. For example, cleavage of *β*-carotene by a DyP from *Lepista irina* resulted in formation of the volatiles *β*-ionone, *β*-cyclocitral, dihydroactinidiolide, and 2-hydroy-2,6,6-trimethylcyclohexanone [41].

## 3. Materials and Methods

### 3.1. Chemicals and Materials

Chemicals were obtained from Sigma-Aldrich (Seelze, Germany), Carl-Roth (Karlsruhe, Germany), or Merck (Darmstadt, Germany) in *p. a.* quality. Enzymes were from Thermo Fisher Scientific (Braunschweig, Germany), if not stated otherwise. PCR primers were obtained from Eurofins MWG Operon (Ebersberg, Germany).

### 3.2. Cultivation of P. sapidus

*P. sapidus* (Deutsche Sammlung von Mikroorganismen und Zellkulturen GmbH, DSMZ, strain no. 2866) was pre-grown on 1.5% (*w*/*v*) agar plates with standard nutrient liquid (SNL) medium and maintained at 4 °C until use [39]. For pre-cultivation, 1 cm^2^ of grown agar was transferred to 100 mL SNL medium and homogenized using an Ultraturrax homogenizer (ART Prozess- & Labortechnik, Müllheim, Germany). The pre-cultures were incubated for 5 days at 150 rpm and 24 °C. Afterwards, 6.5 g of pre-grown mycelium was used to inoculate 250 mL SNL. The main culture was incubated at 150 rpm and 24 °C. After six days, the mycelium was separated from the culture supernatant by centrifugation (5000× *g*, 4 °C, 15 min) and lyophilized as described elsewhere [42].

### 3.3. Purification Strategy

Ten g of lyophilized mycelium were re-suspended in 400 mL buffer A (50 mM Bis-Tris, pH 6.0, 1 M (NH_4_)_2_SO_4_) and extracted for 1 h at 4 °C in horizontal position in an orbital shaker. Insoluble components were removed by centrifugation (5000× *g*, 4 °C, 15 min) followed by filtration (PES filter, 0.45 µm, Merck). Subsequently, 80 mL filtered supernatant were applied on a Phenyl Sepharose fast flow column (20 mL, GE Healthcare Bio-Sciences AB, Uppsala, Sweden) pre-equilibrated with buffer A. After the column was washed with buffer A, the active enzyme was eluted with a linear gradient (130 mL, 100–0% buffer A) with 100% distilled water at a constant flow rate of 2 mL/min. Active fractions were pooled, desalted and concentrated by ultrafiltration (3 kDa cut off, polyethersulfone (PES), Sartorius, Göttingen, Germany). Concentrate (20 mL) was diluted two times with 20 mM sodium acetate pH 4.0 (buffer B) and loaded onto three linked HiTrap SP Sepharose columns (1 mL, GE Healthcare Bio-Sciences AB) pre-equilibrated with buffer B. Proteins were eluted with a stepwise ionic strength gradient (0, 20, 100% buffer C: 20 mM sodium acetate pH 4.0, 1 M NaCl) with 100% buffer C at a constant flow rate of 1 mL/min.

### 3.4. Gel Electrophoresis

SDS-PAGE analysis was performed as described elsewhere [43]. Semi-native PAGE was performed under non-denaturing conditions using 12% gels. For this, samples were prepared with a native loading buffer (without DTT and without 2% (*w*/*v*; 6.9 mM) SDS) and gel electrophoresis was performed at 10 mA per gel and 4 °C. Gels were stained with 0.5 mM ABTS (dissolved in 100 mM sodium acetate buffer pH 3.5 or 4.5) in the presence of 100 µM hydrogen peroxide for detection of peroxidases. For deglycosylation, samples were treated with 1 µL (500 U) endoglycosidase H (EndoH, New England BioLabs, Ipswich, MA, USA) in 20 µL for 2 h at 37 °C before gel electrophoresis.

### 3.5. Isoelectric Focussing

Analytical isoelectric focusing polyacrylamide gel electrophoresis was performed on a HPE^TM^ BlueHorizon^TM^ system (Serva Electrophoresis GmbH, Heidelberg, Germany) using Servalyt^TM^ Precotes^TM^ Precast Gels (Serva Electrophoresis GmbH) with an immobilized pH gradient of pH 3 to 10. To determine the isoelectric points of the enzymes, marker proteins (IEF-Marker 3-10, Serva Electrophoresis GmbH) were used. Gels were stained with Coomassie Brilliant Blue, or for specific visualization of peroxidases, with 1% (*w*/*v*; 9.2 mM) phenylendiamine and 1% (*w*/*v*; 10.6 mM) urea peroxide (dissolved in 100 mM sodium acetate buffer pH 3.5) at RT until a yellow band was observed.

### 3.6. Peptide Mass Fingerprinting

Protein bands were excised from SDS gels, dried, and tryptically hydrolysed. The resulting peptides were extracted and purified according to standard protocols and the amino acid sequence was analyzed with electrospray ionization-tandem mass spectrometry (ESI-MS/MS) using a maXis quadrupole time of flight (QTOF) mass spectrometer (Bruker, Bremen, Germany) as described previously [43,44]. The obtained partial sequences of PsaPOX and of the oxidase were used for a similarity search against public databases (NCBI BlastP).

### 3.7. cDNA Synthesis and Gene Amplification

Isolation of total RNA from mycelium of *P. sapidus* at culture day six and cDNA synthesis were performed as described previously [13] using the primer 5′-AAGCAGTGGTATCAACGCAGAGT ACGCTTTTTTTTTTTTTTTTTTT-3′ for reverse transcription. Specific primers for gene amplification were deduced from the ORF-start (P1: 5′-ATGACTACACCTGCACCACCCCTCGACCTC-3′) and -stop (P2: 5′-TCAAGCAGAGATTGGAGCTTGGGTSWGAGGA-3′) region of the homologous peroxidase of *Pleurotus ostreatus* PC15 (GenBank accession no. KDQ22873.1). PCRs were performed with Phusion High-Fidelity DNA Polymerase and the Master Cycler gradient (Eppendorf, Hamburg, Germany) as described elsewhere [45]. The cycler program was as follows: denaturation for 2 min at 98 °C, 35 cycles at 98 °C for 1 min, 62 °C for 30 s and 72 °C for 90 s, and a final elongation at 72 °C for 10 min. Analysis of PCR products, ligation, transformation in *Escherichia coli*, colony PCR, and sequencing were performed as described by Behrens et al. [13]. Translation of DNA sequences was performed using SnapGene^®^ (GSL Biotech LLC, Chicago, IL, USA). Sequence homology was examined using BLAST [46]. Alignments were produced by ClustalOmega [47].

### 3.8. Heterologous Expression of PsaPOX in Komagataella pfaffii

The gene of PsaPOX was amplified with a C-terminal 6x His tag using the primers PsaPOX_fw 5′-AAAAAGAATTCatgactacacctgcaccacccctcgacctcaacaa-3′ and PsaPOX_rev 5′-atatatGCGGCCGC tcaGTGGTGATGGTGATGATGggtagagatcggagcctgggcctg-3′ (underlined are the EcoRI and NotI restriction sites, respectively; lower cases represent parts of the coding *PsaPOX*). In addition, it was inserted in frame with the *Saccharomyces cerevisiae α*-factor secretion signal sequence into the *K. pfaffii* pPIC9 expression vector (Invitrogen, Karlsruhe, Germany). The resulting expression construct pPIC-PsaPOX-His was transformed into *E. coli* TOP10 for vector propagation, isolated (NucleoSpin, Macherey-Nagel, Düren, Germany), linearized with PmeI, and used for transformation of *K. pfaffii* GS115 according to a standard protocol [48]. The linearized empty vector was transformed in the same way and served as negative control. Forty-eight transformants were tested for peroxidase activity after selection according to their ability to grow on histidine-deficient agar plates in 96-well plates for 120 h, as described elsewhere [49]. Gene expression was induced by daily addition of 1% (*v*/*v*) methanol.

### 3.9. His-Tag Purification of Recombinant PsaPOX

For purification of the His-tag labelled recombinant enzyme from *K. pfaffii* culture supernatant, Ni-NTA affinity chromatography was used according to Nieter et al. [50].

### 3.10. Biotransformation

Transformation of *trans*-anethole was carried out in 4 mL gas tight glass vials in horizontal position at a shaking rate of 200 rpm for 16 h at RT in the absence of light. Reaction mixtures contained 30 mg *P. sapidus* lyophilisate or 100 µL liquid sample buffered in Bis-Tris (50 mM, pH 6) with or without addition of 1 mM manganese sulfate in a total volume of 1 mL and 1 µL (6.7 mM) *trans*-anethole. Blanks (chemical: without lyophilisate or liquid sample; biological: with heat inactivated mycelium (1 h at 95 °C)) were performed the same way. All experiments were performed as duplicates. After incubation, *trans*-anethole and its conversion product *p*-anisaldehyde were extracted with 1 mL hexane containing 100 mg/L (1 mM) cyclohexanol as internal standard (IS). The organic phase was dried with anhydrous sodium sulfate and subsequently analyzed by gas chromatography (GC). GC measurements were performed with an Agilent 7890 instrument equipped with a DB-WAX UI column (30 m × 0.32 mm, 0.25 µm, Agilent, Santa Clara, CA, USA), a split/splitless injector port (1:5) and a flame ionization detection (FID) system. Hydrogen was used as carrier gas at a constant flow rate of 2.1 mL per minute. One µL sample was injected via an autosampler and measured using the following method: 40 °C (3 min), a temperature increase of 10 °C per minute until 230 °C and a final hold time of 10 min. The *trans*-anethole and *p*-anisaldehyde were semi-quantified referring to the area of the internal standard. Biotransformation products were identified using standards and comparison of retention indices with literature.

### 3.11. Enzyme Activities

Total peroxidase activity was determined photometrically (EON^TM^ High Performance Microplate Spectrophotometer, BioTek Instruments GmbH, Bad Friedrichshall, Germany) by monitoring the oxidation of ABTS in the presence of hydrogen peroxide at 420 nm (ε_420_ = 3.6 × 10^4^ M^−1^ cm^−1^) and 30 °C for 10 min. For this, the samples were mixed with sodium acetate buffer (100 mM, pH 4.0 or pH 3.5), 0.1 mM hydrogen peroxide, and 0.5 mM ABTS in a total volume of 300 µL. One unit of enzyme activity was defined as 1 µmol substrate oxidized per minute under the experimental conditions.

To determine manganese peroxidase activity, samples were mixed with manganese sulfate (1 mM), malonate buffer (100 mM, pH 3.5), and hydrogen peroxide (0.1 mM) in a total volume of 300 µL. Mn^3+^ formation was monitored photometrically at 270 nm (ε_270_ = 1.16 × 10^4^ M^−1^ cm^−1^) and 30 °C for 30 min. One unit of enzyme activity was defined as 1 µmol Mn^3+^ per minute released by manganese peroxidases at the given conditions.

Decolorization of Reactive blue 19 (RB19, 150 µM) and Reactive black 5 (RB5, 80 µM) by PsaPOX (1 U/L; 0.25 mg/L) was tested. The respective anthraquinone dye and the enzyme was incubated in the presence of 100 µM hydrogen peroxide, 25 mM manganese sulfate, and 100 mM sodium acetate buffer pH 3.5 in a total volume of 300 µL at 40 °C for 20 min. Decolorization was monitored photometrically at 595 nm (RB19; ε_595_ = 1.0 × 10^4^ M^−1^ cm^−1^) or 598 nm (RB5; ε_598_ =3.0 × 10^4^ M^−1^ cm^−1^). One unit of enzyme activity was defined as 1 µmol dye degraded per minute at the given conditions.

All enzyme assays were performed as triplicates. Blanks were carried out with water instead of enzyme and by omission of hydrogen peroxide.

### 3.12. Biochemical Characterization of PsaPOX

Effects of pH and temperature on peroxidase activity of PsaPOX (0.25 mg/L) were analyzed with ABTS as substrate as described above (see “4.11”). Relative activities were normalized to the highest activity and residual activities to the initial activity prior incubation. The pH optimum was determined using Britton-Robinson buffer [51] in a range of pH 2.0–9.5 instead of sodium acetate buffer. For determination of the temperature optimum the activity assay was performed at different temperatures (20–90 °C) at pH 3.5, whilst for analysis of the temperature stability the enzyme was incubated for 1 h at 20–90 °C prior enzyme activity measurement at pH 3.5 and 40 °C. For the analysis of pH-stability PsaPOX was incubated in Britton-Robinson buffer from pH 2.0 to 9.5 for 1 h at RT before the peroxidase activity was examined at pH 3.5 and 40 °C. 

Hydrogen peroxide as well as Mn^2+^ dependency of PsaPOX were determined for PsaPOX by evaluation of peroxidase activity as described above (“4.11”) with changing hydrogen peroxide and manganese sulfate concentrations (H_2_O_2_: 0–1 mM H_2_O_2_, without addition of MnSO_4_; Mn^2+^: 100 µM H_2_O_2_, 0–100 mM MnSO_4_) at optimal pH and thermal conditions. Kinetic constants of PsaPOX were calculated for Mn^2+^ and ABTS (0–300 µM ABTS in the presence of 100 µM H_2_O_2_ and 25 mM MnSO_4_) by SigmaPlot 12.5 (Systat Software Inc., Chicago, IL, USA) with nonlinear regression. Protein concentrations were determined according to Lowry et al. [52] using bovine serum albumin as standard.

### 3.13. Alkene Cleavage Activity of PsaPOX

The purified recombinant PsaPOX was used for transformation of *trans*-anethole as mentioned above (“4.10”) to confirm alkene cleavage activity. For this, 1 U/mL (0.25 mg/mL) of enzyme was used for biotransformation. Biotransformation was performed with 100 mM sodium acetate buffer pH 3.5 in the presence of 100 µM hydrogen peroxide and 25 mM manganese sulfate at RT for 16 h. Biotransformation of the alkenes methyl isoeugenol (6.7 mM), *α*-methylstyrene (6.7 mM), and piperine (0.7 mM) was tested accordingly. Blanks were performed without enzyme (chemical blank) or with heat inactivated enzyme (1 h at 95 °C, biological blank). The determined product concentrations for the blanks were subtracted from the concentrations yielded for the reaction with the active enzyme to calculate the enzymatically generated product concentration. For carotene degradation, a *β*-carotene emulsion was prepared according to Linke et al. [43]. 7% (*v*/*v*) of *β*-carotene emulsion or annatto (Chr. Hansen, Nienburg, Germany, Prod. No. 240569), 100 µM hydrogen peroxide, 25 mM manganese sulfate, 100 mM sodium acetate pH 3.5, and 1 U/L (0.25 mg/L) PsaPOX in a total volume of 300 µL was incubated at 40 °C for 20 min. Alkene cleavage of both substrates was measured photometrically as extinction decrease at 455 nm.

### 3.14. Detection of Hydrogen Peroxide

For the detection of H_2_O_2_, 75 µL IEX fraction, 50 mM Bis-Tris pH 6.0, 6.7 mM *trans*-anethole, 10 U/mL HRP (Sigma Aldrich), and 0.5 mM *o*-dianisidine in a total volume of 300 µL were incubated at RT for 1 h. In the presence of hydrogen peroxide, formation of a red-brown reaction product occurred. Blanks were performed with 50 mM Bis-Tris pH 6.0 instead of IEX fraction.

### 3.15. Sequence Accession Numbers

The nucleotide sequence of the *PsaPOX* gene has been deposited in the GenBank database under accession number MT043310.

## 4. Conclusions

A DyP-type peroxidase of *P. sapidus* with alkene cleavage activity as well as the corresponding gene were identified and the gene was heterologously expressed in *Komagataella pfaffii*. The PsaPOX possessed typical sequence motifs, structural topology, and catalytic residues as described for DyPs, even though the decolorization of the anthraquinone Reactive blue 19, a common reaction for DyPs, was not observed. A non-canonical Mn^2+^-oxidation site on the protein surface was detected, which allows PsaPOX to oxidize Mn^2+^. After biochemical characterization, the alkene cleavage activity of PsaPOX towards different aryl alkenes was confirmed by biotransformation. PsaPOX is the first described DyP-type peroxidase with such an activity. In addition, bleaching of *β*-carotene and annatto was determined. The results for the alkene cleavage underline the potential of the PsaPOX as biocatalyst for the generation of aromatic aldehydes with olfactory properties, such as *p*-anisaldehyde, veratraldehyde, or acetophenone, which are used in the fragrance and flavor industry [1]. Improvement of the conversions and product yields may be accomplished by protein engineering, as has been shown for the alkene cleaving manganese-dependent Cupin TM1459 from *Thermotoga maritima* [53]. Another application beyond aroma production could be carotene bleaching of whey or wheat dough.

## Figures and Tables

**Table 1:** Michaelis constants (*K*_m_), catalytic constants (*k*_cat_), and catalytic efficiencies (*k*_cat_/*K*_m_) for PsaPOX using ABTS, Mn^2+^, Reactive blue 19 (RB19), and Reactive black 5 (RB5) as substrate. Values are the average of triplicate experiments with indication of standard deviations.

Substrate	*K*_m_ (µM)	*k*_cat_ (s^−1^)	*k*_cat_/*K*_m_ (s^−1^ mM^−1^)
ABTS	37 ± 4	6.8 ± 0.2	184 ± 5
Mn^2+^	1025 ± 79	7.2 ± 0.1	7 ± 0.1
RB19	n. d.	-	-
RB5	n. d.	-	-

n. d.: no activity was detected.

## Supplementary Materials

The following are available online, Figure S1: Structural homology model of PsaPOX, Figure S2: Detection of hydrogen peroxide in the IEX fraction with *o*-dianisidine and HRP in the presence of *trans*-anethole after 1 h of incubation at pH 6.0 and RT, Figure S3: Alignment of the hypothetical protein (KDQ29984.1) from *P. ostreatus*, which was the best hit for the 75 kDa band of the IEX fraction by a homology search against the public database NCBI, and other members of the GMC oxidoreductase family, Figure S4: Stability of PsaPOX during biotransformation of *trans*-anethole over 16 h, Table S1: *p*-Anisaldehyde concentration after biotransformation of *trans*-anethole with different basidiomycetes, Table S2: *p*-Anisaldehyde concentration after biotransformation of *trans*-anethole with the active IEX fraction in the presence or absence of Mn^2+^ and/or H_2_O_2_ for 16 h at RT, Table S3: *p*-Anisaldehyde concentration after bioconversion of *trans*-anethole by recombinant PsaPOX with and without addition of H_2_O_2_ and Mn^2+^ for 16 h at RT, Table S4: *p*-Anisaldehyde concentration after biotransformation of *trans*-anethole, (*E*)-methyl isoeugenol, and *α*-methylstyrene by recombinant PsaPOX (1 U/mL) in the presence of 100 µM H_2_O_2_ and 25 mM MnSO_4_ for 16 h at pH 3.5 and RT.
