# Supplementary material for: A DyP-Type Peroxidase of Pleurotus sapidus with Alkene Cleaving Activity
Source: Molecules. 2020 Mar 27;25(7):1536. doi: 10.3390/molecules25071536 (PMC7181223; doi:10.3390/molecules25071536)
Supplement: Supplementary file 1 [file molecules-25-01536-s001.pdf]

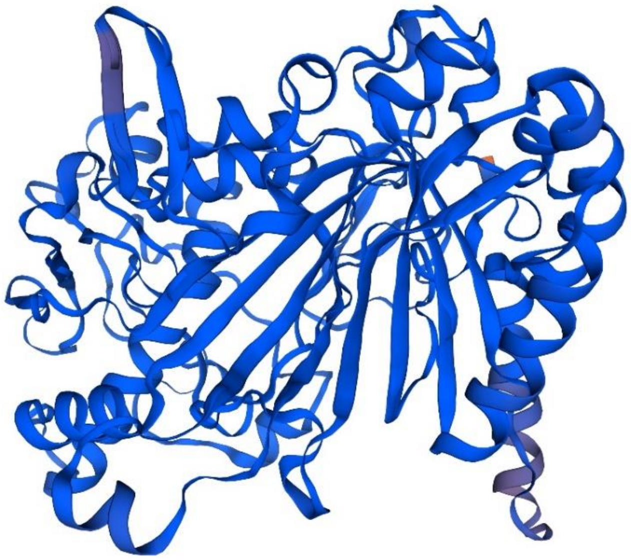

Figure 1. Structural homology model of PsaPOX. The model was generated with the SWISS-MODEL server using the X-ray crystal structure of *Pleos*-DyP4 (PDB-ID 6fsk).

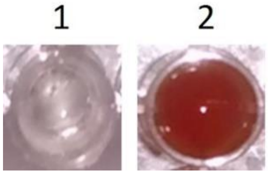

Figure 2. Detection of hydrogen peroxide in the IEX fraction with *o*-dianisidine and HRP in the presence of *trans*-anethole after 1 h of incubation at pH 6.0 and RT. 1: blank (buffer instead of IEX fraction); 2: IEX fraction. *o*-Dianisidine was oxidized to a red-brown colorant by HRP in the presence of H_2O_2.

```
KDQ29984.1      mlhqsypyltshlawlpltsasafvywrgidlplifpsrlsrhnlragrrmihdtnrgvqgpdwr    65
KDQ29972.1      -----------------------------------------------------mhlflr--lliacv    12
ESK85927.1      ------------------------------------------------------------mfsiyp    6
KLO09144.1      -------------------------------------------------------mk-ail---s    6
THU81532.1      -----------------------------------------------------mt---vk-flvaacv    11

KDQ29984.1      --glrssiatsstydyviigggtaglavanrlsedplvsiavieagpnaehlrevfipglf---g    125
KDQ29972.1      --glrssiatssaydyviigggtaglavasrlsedpsvsivvieagpnaehlpevfipglf---g    72
ESK85927.1      avivlsllhsafsydyvvigggtagltvasrlaeddsvqvavieaganaehrpdvfipgmlgqig    71
KLO09144.1      ylfvlqlalrsvafdyvivgggtagltvasrlsedpsvtvavieagpnaenlpevfvpgli---g    68
THU81532.1      aalapvanvlassydyiiigggtggltvasrlaedksnqvlvleagpnaeglqevfvpgli---g    73
                         : ::**:::****.**:**.**:**    : *:*** ***   :**:**::   *

KDQ29984.1      sgqalttlnwaykttpqpmlnnrslvvnagrvlggsttinamlfpragraqydawaelnrdaswg    190
KDQ29972.1      sgqafttlnwaynttpqpmlnnrslvvnagrvlggsttinamlfpragraqydawaelnrdaswg    137
ESK85927.1      vgtkppelnwayktvpqthlngrvltvnagralggstvinamifprakkeqydawgilnndtswt    136
KLO09144.1      tgqsfttlnwayrtvpqvnlnsrnltvnagkalggstiinsmifpraekeqydvwgalnndsswt    133
THU81532.1      tgqsfttlnwayrtapqenlnnrtltvnagkalggstiinsmifpraekaqydawgtlnndsswt    138
                 *     *****.*.**  **.* *.****:.***** **:*:**** : ***.*. **.*:**

KDQ29984.1      wdallpffrrservtppdafqvnagsvtyvpevhgsa-sdgrvkvgypniffeqstlwrqaavav    254
KDQ29972.1      wdallpffrrservtppdafqvneggityvpevhgsa-sdgrvkvgypniffeqstlwrqaavav    201
ESK85927.1      weallpffkrsesftvpndfqasn-garydpqfhgfd---gslkvgfpnyffpqemlwqntsigl    197
KLO09144.1      wdallpffknseiftppnafqsas-gvrfdpsvhgfs---grvhvgfpnfffeqsqlwvktsegl    194
THU81532.1      wdallpffkkseiftppnefqrdn-gatfvrdvhgfnesegrvkvgfpnfffpqstlwrqtsmgl    202
                *:******:.** .* *: **     .  :  ..**     * ::**:** ** *. ** ::: .:

KDQ29984.1      gfpgerdlasgdphgvgvspesidvtnytrcsaacayytpfasrpnfnvitnatvtrivwdtpsp    319
KDQ29972.1      gfpgerdlasgdphgvgvspesidvtnytrcsaacayytpfasrpnfnvitnatvtrvvwdtpss    266
ESK85927.1      gfpaatdlsngephavgpspfsidainqtrcsaacafytpfadrpnftiitnanvtriiwsesen    262
KLO09144.1      gfpaspdlangdphavgvapdslnaanntrcsaacayftpfaerpnftvflnatvsriiwdsnst    259
THU81532.1      gfpaspdlangsphavgvapdsldaanntrcsaacayftpfaeqpnftvltnvtvtriiwtasaq    267
                ***.  **:.*.**.** :* *::. * ********::****.:***.:: *..*:*::*

KDQ29984.1      gshsnegqrnlraiavefvragsnr-tesvtvrrevvvsagtigtprvfelsgignssiiraagv    383
KDQ29972.1      gshsdksqrnlraiavefvragsnr-tesvtvrrevvvsagtigtpklfelsgignssiiraagv    330
ESK85927.1      n-------sslaatgveyvtnnetm---tlevtgevilsagtigspkilelsgignstilraagv    317
KLO09144.1      an-----agstkaiaveyiasngttlqa--sverevivsagtigspkvlelsgvgnatiltaagv    317
THU81532.1      nd------stltasgveyidslnqtsrailsrkgevivsagtigspkilelsgvgnstilqaagi    326
                         .  * .**::                  **::******:*:::****:**::*: ***:

KDQ29984.1      npvlelpsvgenlag--------------------------------------lltsfpgslgl    409
KDQ29972.1      npvlelpsvgenlagef------------------------------------clltsfpgslgl    358
ESK85927.1      eplldlptvgenla---------------------------------------gmyaavsktlgi    342
KLO09144.1      qpqvnlstvgenladhvhnwvnsftsfnltkdl-lnnatflaeqlelwnktrtgllsaaprslgi    381
THU81532.1      spaldlptvgenladhvhswanaftnisltkdalllntdfaqqqldlwfknrtglysaaprslgi    391
                .* ::* :******                                        : ::   :**:

KDQ29984.1      pgplnifndtrilptllqeakanlshfahlfangnallekgiraqheivlrsyaddvalplens-    473
KDQ29972.1      pgplnifnntgilptllqeakanlthfahlfangnallekgiraqheivlrsyaddvalplensl    424
ESK85927.1      tapldvvsqsq-ldtllltqaesnlshyasqfsngnpdlargieaqhrlafslyqenqqlcveln-    406
KLO09144.1      aapsnvfnqsq-ltalvtqsranltataisfsngnadlakgiaaqldhalslyaedkelplemn-    444
THU81532.1      aapsdvfeese-fnrllnqseeslsrfasefsngnpqlakgiesqyrialslyrqnenlplemn-    454
                 .* ::..::  :  *: ::. .*:  *  *:***  * :** :*   .:  * ::  * :* .

KDQ29984.1      ------lfpgyaga--aadrpqrnyttimnvlyaplsrgrthitssdirtapvvdpayymhplda    530
KDQ29972.1      caallsyfpgyaga--aadrpqrnyttimnvlyaplsrgrthitssdirtapvvdpayymhplda    487
ESK85927.1      ------ldagyvgptplesrplrnfttvstilyaplargrshisssdpftvplvdpaywahpmdv    465
KLO09144.1      ------lepgysgptplavrpnrtfttintvlyaplsrgrthitssspsvppavdpnyyahpmdi    503
THU81532.1      ------lepgysgptafadrparnytainsvlysplsrgrthissssplaapvvdpaywahpldv    513
                         ** *      ** *.:*:: .:**:**:***:**:**.  . * *** *: **:*

KDQ29984.1      athaaginlarktlitppmdsiylgefepgkdvtspqdissvlraaivssdnhvtgtmammpkel    595
KDQ29972.1      athaaginlarrtlitppmdsiylgefepgkdvtspqdissvlraaivssdnhvtgtmammpqel    552
ESK85927.1      aihvggiqlakkmlrsppldsiydgefepgve--sdkeienwlrgn-vtsdnhvtgtlsmlprel    527
KLO09144.1      aahiggirlarrmltspplgdsflgefepgaekvsdaqigswlran-atsdnhetgtaammprel    567
THU81532.1      aiqvagiklarkmlqssplsstyegefepgtdketdaeieewlrgv-vasdnhevgslamlpkdm    577
                * : .**.**:: * : *:.. : ****** :  :  :* . **.  .:**** .*: :*:*:::

KDQ29984.1      ggvvdtelrvygignvrvadasiipipvsahtsstvymigeraadlikhsrspl-----------    649
KDQ29972.1      ggvvdtelrvygienvrvadasiipipvsahtvgplrllithladlqpvvdclhdwraresilwv    622
ESK85927.1      ggvvdtqlkvygtmnvrvvdasvipfpvsahtsstvymigeraadiirksrgn------------    580
KLO09144.1      ggvvdtnlkvygtanvrvvdasiipfpisahisstvyaigekvrqsd-----------------    614
THU81532.1      ggvvdtellvygtsnvrvvdasvipfpisahlsssvymigeraadiikrn---------------    627
                ******:* ***  ****.***:**:*:***  . :  :  :  :

KDQ29984.1      ---------------------------                                          649
KDQ29972.1      lllaafltrvlrlqisssivdihcnhl                                          644
ESK85927.1      ---------------------------                                          580
KLO09144.1      ---------------------------                                          614
THU81532.1      ---------------------------                                          627
```

Figure 3. Alignment of the hypothetical protein (KDQ29984.1) from *P. ostreatus*, which was the best hit for the 75 kDa band of the IEX fraction by a homology search against the public database NCBI, and other members of the GMC oxidoreductase family. KDQ29972.1: hypothetical protein from *P. ostreatus* (92% identity to KDQ29984.1); ESK85927.1: glucose oxidase from *Moniliophthora roreri* (57% identity to KDQ29984.1); KLO09144.1: alcohol oxidase from *Schizopora paradoxa* (56% identity to KDQ29984.1); THU81532.1: alcohol oxidase from *Dendrothele bispora* (55% identity to KDQ29984.1). Asterisks indicate conserved residues, colons equivalent residues and dots partial residue conservation. Peptides identified by protein sequencing are underlined. Alignment was performed with Clustal Omega (European Bioinformatics Institute, Hinxton, UK).

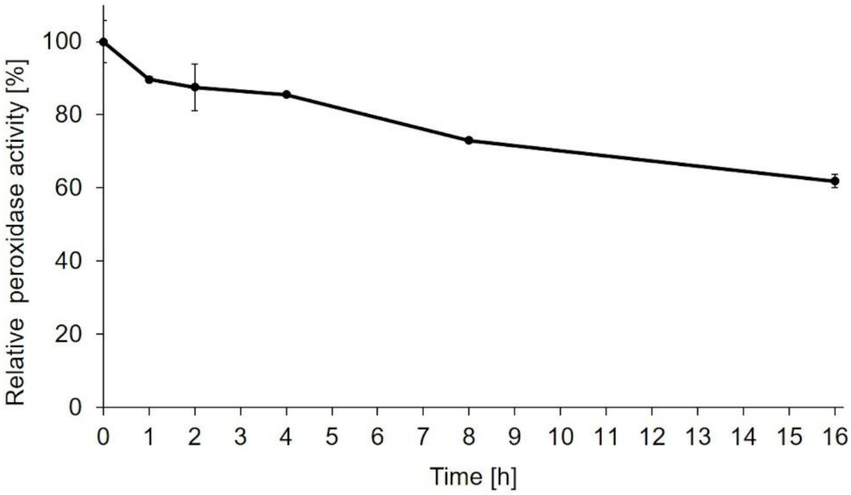

Figure 4. Stability of PsaPOX during biotransformation of *trans*-anethole over 16 h. The reaction was performed in the presence of 100 μM H_2O_2 and 25 mM $MnSO_4$ in 100 mM sodium acetate buffer pH 3.5 at RT. Relative peroxidase activity [%] was defined as the percentage of activity detected with respect to the starting activity. Values are the average of triplicate experiments with standard deviations shown as error bars.

Table 1. *p*-Anisaldehyde concentration after biotransformation of *trans*-anethole with different basidiomycetes.

strain	*p*-anisaldehyde concentration [mM]	origin
Pleurotus sapidus	5.36	DSMZ, strain no. 2866
Pleurotus eryngii	4.07	CBS, strain no. 613.91
Pleurotus ostreatus	3.74	DSMZ, strain no. 1020
Pleurotus ostreatus var. *florida*	2.00	Laboratory collection
Pleurotus sajor-caju	1.14	DSMZ, strain no. 5339
Pleurotus pulmonarius	1.04	DSMZ, strain no. 5331
*Trametes versicolor**	0.96	Laboratory collection
Trametes suaveolens	0.62	DSMZ, strain no. 5237
Pleurotus lampas	0.79	CBS, strain no. 323.49
Lentinus lepideus	0.29	CBS, strain no. 450.79
Wolfiporia cocos	0.18	CBS, strain no. 279.55
Gloeophyllum odoratum	0.18	CBS, strain no. 444.61
Piptoporus soleniensis	0.17	CBS, strain no. 492.76
Schizophyllum commune	0.10	DSMZ, strain no. 1024
Gloephyllum trabeum	0.00	DSMZ, strain no. 3087
Phanerochaete chrysosporium	0.00	DSMZ, strain no. 1547

* Two variants of the strain were tested for the bioconversion. The results for the strain with higher *p*-anisaldehyde concentrations are presented.

DSMZ: Deutsche Sammlung von Mikroorganismen und Zellkulturen GmbH, CBS: Westerdijk Fungal Biodiversity Institute

Table 2. *p*-Anisaldehyde concentration after biotransformation of *trans*-anethole with the active IEX fraction in the presence or absence of Mn^{2+} and/or H_2O_2 for 16 h at RT. The *p*-anisaldehyde concentrations are the average of duplicate experiments with indication of standard deviations.

$MnSO_4$ concentration [mM]	H_2O_2 concentration [µM]	*p*-anisaldehyde concentration [µM]
0	0	30 ± 1
1	0	450 ± 17
1	50	600 ± 12

Table 3. *p*-Anisaldehyde concentration after bioconversion of *trans*-anethole by recombinant PsaPOX with and without addition of H_2O_2 and Mn^{2+} for 16 h at RT. The *p*-anisaldehyde concentrations are the average of duplicate experiments with indication of standard deviations.

$MnSO_4$ concentration [mM]	H_2O_2 concentration [µM]	*p*-anisaldehyde concentration [µM]
0	0	0 ± 0
0	100	189 ± 6
25	100	320 ± 20

Table 4. *p*-Anisaldehyde concentration after biotransformation of *trans*-anethole, (*E*)-methyl isoeugenol, and *α*-methylstyrene by recombinant PsaPOX (1 U/mL) in the presence of 100 µM H_2O_2 and 25 mM $MnSO_4$ for 16 h at pH 3.5 and RT. The values are the average of duplicate experiments with indication of standard deviations.

Substrate	Product	Product concentration active enzyme [µM]	Product concentration inactivated enzyme (control) [µM]
trans-anethole	*p*-anisaldehyde	342 ± 20	22 ± 7
(*E*)-methyl isoeugenol	veratraldehyde	114 ± 12	48 ± 9
α-methylstyrene	acetophenone	24 ± 7	0 ± 0
